# Structural basis for the recognition of SARS-CoV-2 by full-length human ACE2

**DOI:** 10.1126/science.abb2762

**Published:** 2020-03-04

**Authors:** Renhong Yan, Yuanyuan Zhang, Yaning Li, Lu Xia, Yingying Guo, Qiang Zhou

**Affiliations:** 1 Key Laboratory of Structural Biology of Zhejiang Province, Institute of Biology, Westlake Institute for Advanced Study, 18 Shilongshan Road, Hangzhou 310024, Zhejiang Province, China.; 2 School of Life Sciences, Westlake University, 18 Shilongshan Road, Hangzhou 310024, Zhejiang Province, China.; 3 Beijing Advanced Innovation Center for Structural Biology, Tsinghua-Peking Joint Center for Life Sciences, School of Life Sciences, Tsinghua University, Beijing 100084, China.

## Abstract

Angiotensin-converting enzyme 2 (ACE2) is the cellular receptor for severe acute respiratory syndrome–coronavirus (SARS-CoV) and the new coronavirus (SARS-CoV-2) that is causing the serious coronavirus disease 2019 (COVID-19) epidemic. Here, we present cryo–electron microscopy structures of full-length human ACE2 in the presence of the neutral amino acid transporter B^0^AT1 with or without the receptor binding domain (RBD) of the surface spike glycoprotein (S protein) of SARS-CoV-2, both at an overall resolution of 2.9 angstroms, with a local resolution of 3.5 angstroms at the ACE2-RBD interface. The ACE2-B^0^AT1 complex is assembled as a dimer of heterodimers, with the collectrin-like domain of ACE2 mediating homodimerization. The RBD is recognized by the extracellular peptidase domain of ACE2 mainly through polar residues. These findings provide important insights into the molecular basis for coronavirus recognition and infection.

## How SARS-CoV-2 binds to human cells

Scientists are racing to learn the secrets of severe acute respiratory syndrome–coronavirus 2 (SARS-CoV-2), which is the cause of the pandemic disease COVID-19. The first step in viral entry is the binding of the viral trimeric spike protein to the human receptor angiotensin-converting enzyme 2 (ACE2). Yan *et al.* present the structure of human ACE2 in complex with a membrane protein that it chaperones, B^0^AT1. In the context of this complex, ACE2 is a dimer. A further structure shows how the receptor binding domain of SARS-CoV-2 interacts with ACE2 and suggests that it is possible that two trimeric spike proteins bind to an ACE2 dimer. The structures provide a basis for the development of therapeutics targeting this crucial interaction.

*Science*, this issue p. 1444

## Teaser

Insight into how SARS-CoV-2 binds its human receptor could provide a basis for the development of therapeutics.

Severe acute respiratory syndrome–coronavirus 2 (SARS-CoV-2) is a positive-strand RNA virus that causes severe respiratory syndrome in humans. The resulting outbreak of coronavirus disease 2019 (COVID-19) has emerged as a severe epidemic, claiming more than 2000 lives worldwide between December 2019 and February 2020 (*1*, *2*). The genome of SARS-CoV-2 shares about 80% identity with that of SARS-CoV and is about 96% identical to the bat coronavirus BatCoV RaTG13 (*2*).

In the case of SARS-CoV, the spike glycoprotein (S protein) on the virion surface mediates receptor recognition and membrane fusion (*3*, *4*). During viral infection, the trimeric S protein is cleaved into S1 and S2 subunits and S1 subunits are released in the transition to the postfusion conformation (*4*–*7*). S1 contains the receptor binding domain (RBD), which directly binds to the peptidase domain (PD) of angiotensin-converting enzyme 2 (ACE2) (*8*), whereas S2 is responsible for membrane fusion. When S1 binds to the host receptor ACE2, another cleavage site on S2 is exposed and is cleaved by host proteases, a process that is critical for viral infection (*5*, *9*, *10*). The S protein of SARS-CoV-2 may also exploit ACE2 for host infection (*2*, *11*–*13*). A recent publication reported the structure of the S protein of SARS-CoV-2 and showed that the ectodomain of the SARS-CoV-2 S protein binds to the PD of ACE2 with a dissociation constant (*K*_d_) of ~15 nM (*14*).

Although ACE2 is hijacked by some coronaviruses, its primary physiological role is in the maturation of angiotensin (Ang), a peptide hormone that controls vasoconstriction and blood pressure. ACE2 is a type I membrane protein expressed in lungs, heart, kidneys, and intestine (*15*–*17*). Decreased expression of ACE2 is associated with cardiovascular diseases (*18*–*20*). Full-length ACE2 consists of an N-terminal PD and a C-terminal collectrin-like domain (CLD) that ends with a single transmembrane helix and a ~40-residue intracellular segment (*15*, *21*). The PD of ACE2 cleaves Ang I to produce Ang-(1-9), which is then processed by other enzymes to become Ang-(1-7). ACE2 can also directly process Ang II to give Ang-(1-7) (*15*, *22*).

Structures of the claw-like ACE2-PD alone and in complex with the RBD or the S protein of SARS-CoV have revealed the molecular details of the interaction between the RBD of the S protein and PD of ACE2 (*7*, *8*, *23*, *24*). Structural information on ACE2 is limited to the PD domain. The single transmembrane (TM) helix of ACE2 makes it challenging to determine the structure of the full-length protein.

ACE2 also functions as the chaperone for membrane trafficking of the amino acid transporter B^0^AT1, also known as SLC6A19 (*25*), which mediates uptake of neutral amino acids into intestinal cells in a sodium-dependent manner. Mutations in B^0^AT1 may cause Hartnup disorder, an inherited disease with symptoms such as pellagra, cerebellar ataxia, and psychosis (*26*–*28*). Structures have been determined for the SLC6 family members *d*DAT (*Drosophila* dopamine transporter) and human SERT (serotonin transporter, SLC6A4) (*29*, *30*). It is unclear how ACE2 interacts with B^0^AT1. The membrane trafficking mechanism for ACE2 and B^0^AT1 is similar to that of the LAT1-4F2hc complex, a large neutral–amino acid transporter complex that requires 4F2hc for its plasma membrane localization (*31*). Our structure of LAT1-4F2hc shows that the cargo LAT1 and chaperone 4F2hc interact through both extracellular and transmembrane domains (*32*). We reasoned that the structure of full-length ACE2 may be revealed in the presence of B^0^AT1.

Here, we report cryo–electron microscopy (cryo-EM) structures of the full-length human ACE2-B^0^AT1 complex at an overall resolution of 2.9 Å and a complex between the RBD of SARS-CoV-2 and the ACE2-B^0^AT1 complex, also with an overall resolution of 2.9 Å and with 3.5-Å local resolution at the ACE2-RBD interface. The ACE2-B^0^AT1 complex exists as a dimer of heterodimers. Structural alignment of the RBD-ACE2-B^0^AT1 ternary complex with the S protein of SARS-CoV-2 suggests that two S protein trimers can simultaneously bind to an ACE2 homodimer.

## Structural determination of the ACE2-B^0^AT1 complex

**Fig. 1:**
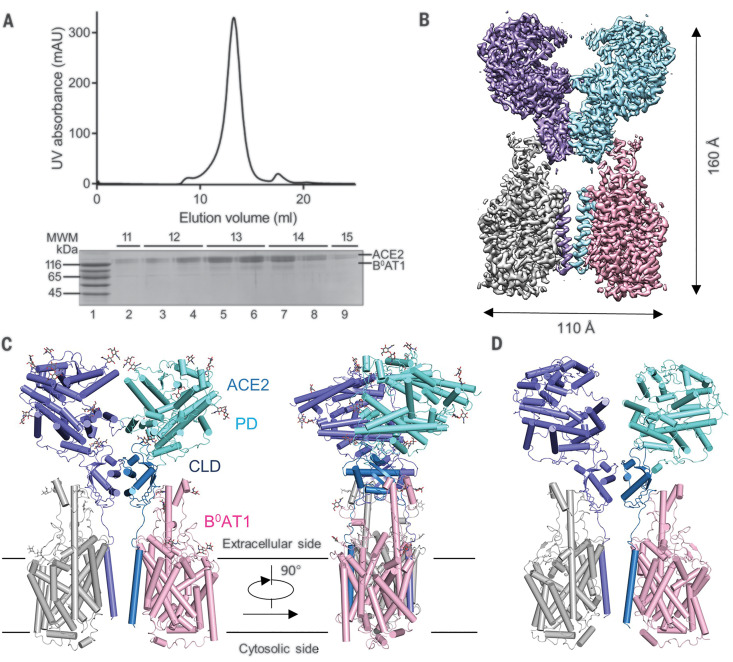
Overall structure of the ACE2-B^0^AT1 complex. (**A**) Representative size exclusion chromatography purification profile of full-length human ACE2 in complex with B^0^AT1. UV, ultraviolet; mAU, milli–absorbance units; MWM, molecular weight marker. (**B**) Cryo-EM map of the ACE2-B^0^AT1 complex. The map is generated by merging the focused refined maps shown in fig. S2. Protomer A of ACE2 (cyan), protomer B of ACE2 (blue), protomer A of B^0^AT1 (pink) and protomer B of B^0^AT1 (gray) are shown. (**C**) Cartoon representation of the atomic model of the ACE2-B^0^AT1 complex. The glycosylation moieties are shown as sticks. The complex is colored by subunits, with the PD and CLD in one ACE2 protomer colored cyan and blue, respectively. (**D**) An open conformation of the ACE2-B^0^AT1 complex. The two PDs, which contact each other in the closed conformation, are separated in the open conformation.

Full-length human ACE2 and B^0^AT1, with Strep and FLAG tags on their respective N termini, were coexpressed in human embryonic kidney (HEK) 293F cells and purified through tandem affinity resin and size exclusion chromatography. The complex was eluted in a single monodisperse peak, indicating high homogeneity (Fig. 1A). Details of cryo-sample preparation, data acquisition, and structural determination are given in the materials and methods section of the supplementary materials. A three-dimensional (3D) reconstruction was obtained at an overall resolution of 2.9 Å from 418,140 selected particles. This immediately revealed the dimer of heterodimers’ architecture (Fig. 1B). After applying focused refinement and C2 symmetry expansion, the resolution of the extracellular domains improved to 2.7 Å, whereas the TM domain remained at 2.9-Å resolution (Fig. 1B, figs. S1 to S3, and table S1).

The high resolution supported reliable model building. For ACE2, side chains could be assigned to residues 19 to 768, which contain the PD (residues 19 to 615) and the CLD (residues 616 to 768), which consists of a small extracellular domain, a long linker, and the single TM helix (Fig. 1C). Between the PD and TM helix is a ferredoxin-like fold domain; we refer to this as the neck domain (residues 616 to 726) (Fig. 1C and fig. S4). Homodimerization is entirely mediated by ACE2, which is sandwiched by B^0^AT1. Both the PD and neck domains contribute to dimerization, whereas each B^0^AT1 interacts with the neck and TM helix in the adjacent ACE2 (Fig. 1C). The extracellular region is highly glycosylated, with seven and five glycosylation sites on each ACE2 and B^0^AT1 monomer, respectively.

During classification, another subset with 143,857 particles was processed to an overall resolution of 4.5 Å. Whereas the neck domain still dimerizes, the PDs are separated from each other in this reconstruction (Fig. 1D and fig. S1, H to K). We therefore define the two classes as the open and closed conformations. Structural comparison shows that the conformational changes are achieved through rotation of the PD domains, with the rest of the complex left nearly unchanged (movie S1).

## Homodimer interface of ACE2

**Fig. 2:**
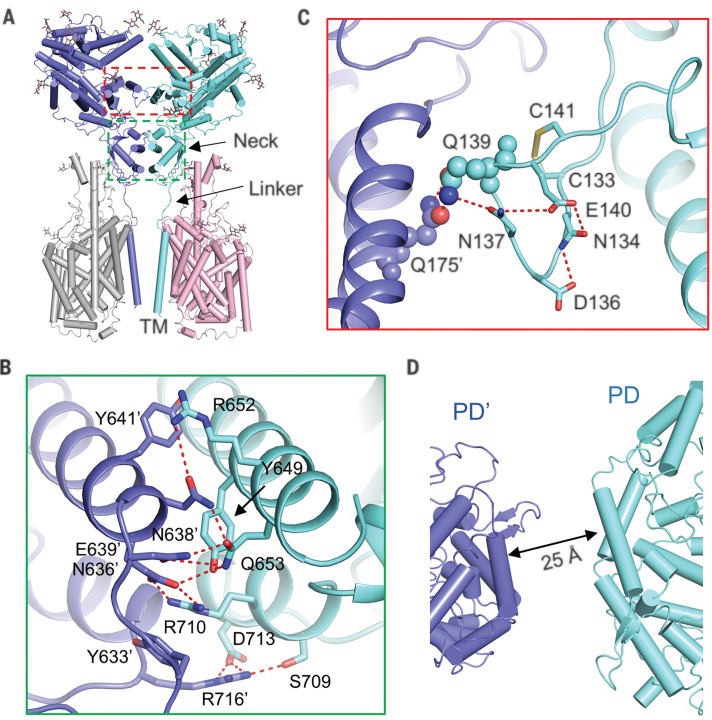
Dimerization interface of ACE2. (**A**) ACE2 dimerizes through two interfaces, the PD and the neck domain. The regions enclosed by the cyan and red dashed lines are illustrated in detail in (B) and (C), respectively. (**B**) The primary dimeric interface is through the neck domain in ACE2. Polar interactions are represented by red dashed lines. (**C**) A weaker interface between PDs of ACE2. The only interaction is between Gln^139^ and Gln^175^^′^, which are highlighted as spheres. The polar residues that may contribute to the stabilization of Gln^139^ are shown as sticks. (**D**) The PDs no longer contact each other in the open state. Single-letter abbreviations for the amino acid residues used in the figures are as follows: C, Cys; D, Asp; E, Glu; F, Phe; H, His; K, Lys; L, Leu; M, Met; N, Asn; Q, Gln; R, Arg; S, Ser; T, Thr; V, Val; and Y, Tyr.

Dimerization of ACE2 is mainly mediated by the neck domain, with the PD contributing a minor interface (Fig. 2A). The two ACE2 protomers are hereafter referred to as A and B, with residues in protomer B followed by a prime symbol. Extensive polar interactions are mapped to the interface between the second (residues 636 to 658) and fourth (residues 708 to 717) helices of the neck domain (Fig. 2B). Arg^652^ and Arg^710^ in ACE2-A form cation-π interactions with Tyr^641^^′^ and Tyr^633^^′^ in ACE2-B. Meanwhile, Arg^652^ and Arg^710^ are respectively hydrogen-bonded (H-bonded) to Asn^638^^′^ and Glu^639^^′^, which also interact with Gln^653^, as does Asn^636^^′^. Ser^709^ and Asp^713^ from ACE2-A are H-bonded to Arg^716^^′^. This extensive network of polar interactions indicates stable dimer formation.

The PD dimer interface appears much weaker, with only one pair of interactions between Gln^139^ and Gln^175^^′^ (Fig. 2C). Gln^139^ is in a loop that is stabilized by a disulfide bond between Cys^133^ and Cys^141^ as well as multiple intraloop polar interactions (Fig. 2C). The weak interaction is consistent with the ability to transition to the open conformation, in which the interface between the neck domains remains the same while the PDs are separated from each other by ~25 Å (Fig. 2D and movie S1).

## Overall structure of the RBD-ACE2-B^0^AT1 complex

To gain insight into the interaction between ACE2 and SARS-CoV-2, we purchased 0.2 mg of recombinantly expressed and purified RBD-mFc of SARS-CoV-2 (for simplicity, hereafter referred to as RBD; mFc, mouse Fc tag) from Sino Biological Inc., mixed it with our purified ACE2-B^0^AT1 complex at a stoichiometric ratio of ~1.1 to 1, and proceeded with cryo-grid preparation and imaging. Finally, a 3D EM reconstruction of the ternary complex was obtained.

**Fig. 3:**
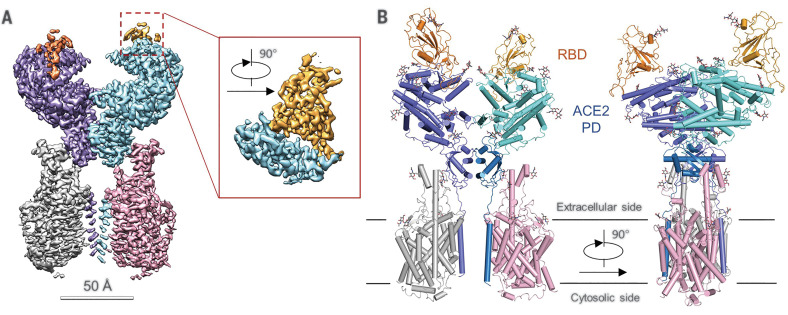
Overall structure of the RBD-ACE2-B^0^AT1 complex. (**A**) Cryo-EM map of the RBD-ACE2-B^0^AT1 complex. The overall reconstruction of the ternary complex at 2.9 Å is shown on the left. The inset shows the focused refined map of RBD. The color scheme is the same as that in Fig. 1B, with the addition of red and gold, which represent RBD protomers. (**B**) Overall structure of the RBD-ACE2-B^0^AT1 complex. The color scheme is the same as that in Fig. 1C. The glycosylation moieties are shown as sticks.

In contrast to the ACE2-B^0^AT1 complex—which has two conformations, open and closed—only the closed state of ACE2 was observed in the dataset for the RBD-ACE2-B^0^AT1 ternary complex. The structure of the ternary complex was determined to an overall resolution of 2.9 Å from 527,017 selected particles. However, the resolution for the ACE2-B^0^AT1 complex was substantially higher than that for the RBDs, which are at the periphery of the complex (Fig. 3A). To improve the local resolution, focused refinement was applied; this allowed us to reach a resolution of 3.5 Å for the RBD, supporting reliable modeling and analysis of the interface (Fig. 3, figs. S5 to S7, and table S1).

## Interface between the RBD and ACE2

**Fig. 4:**
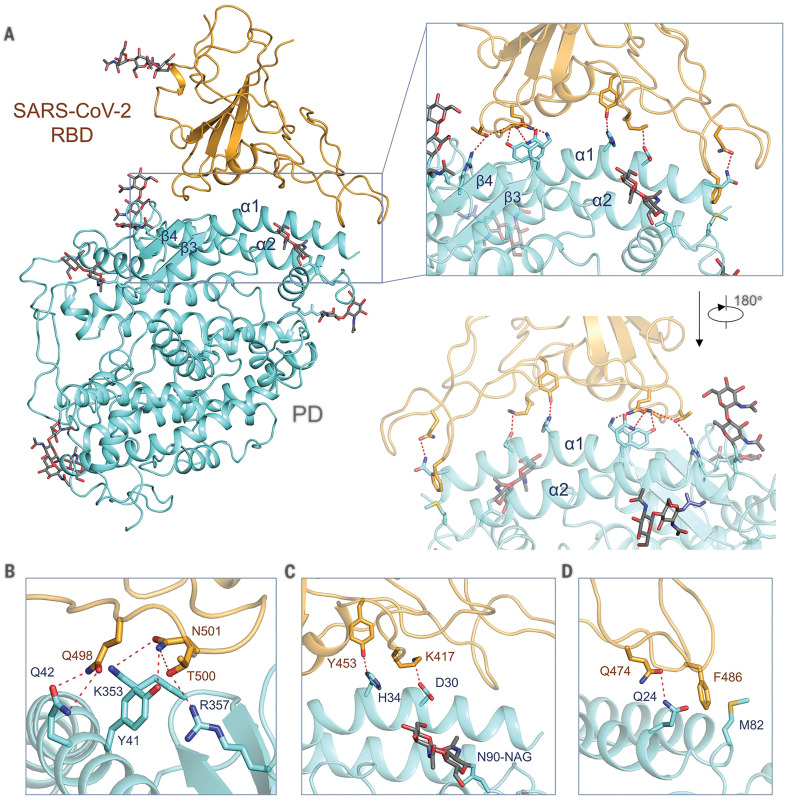
Interactions between SARS-CoV-2-RBD and ACE2. (**A**) The PD of ACE2 mainly engages the α1 helix in the recognition of the RBD. The α2 helix and the linker between β3 and β4 also contribute to the interaction. Only one RBD-ACE2 is shown. (**B** to **D**) Detailed analysis of the interface between SARS-CoV-2-RBD and ACE2. Polar interactions are indicated by red dashed lines. NAG, *N*-acetylglucosamine.

As expected, each PD accommodates one RBD (Fig. 3B). The overall interface is similar to that between SARS-CoV and ACE2 (*7*, *8*), mediated mainly through polar interactions (Fig. 4A). An extended loop region of the RBD spans the arch-shaped α1 helix of the ACE2-PD like a bridge. The α2 helix and a loop that connects the β3 and β4 antiparallel strands, referred to as loop 3-4, of the PD also make limited contributions to the coordination of the RBD.

The contact can be divided into three clusters. The two ends of the bridge interact with the N and C termini of the α1 helix as well as small areas on the α2 helix and loop 3-4. The middle segment of α1 reinforces the interaction by engaging two polar residues (Fig. 4A). At the N terminus of α1, Gln^498^, Thr^500^, and Asn^501^ of the RBD form a network of H-bonds with Tyr^41^, Gln^42^, Lys^353^, and Arg^357^ from ACE2 (Fig. 4B). In the middle of the bridge, Lys^417^ and Tyr^453^ of the RBD interact with Asp^30^ and His^34^ of ACE2, respectively (Fig. 4C). At the C terminus of α1, Gln^474^ of the RBD is H-bonded to Gln^24^ of ACE2, whereas Phe^486^ of the RBD interacts with Met^82^ of ACE2 through van der Waals forces (Fig. 4D).

## Comparing the SARS-CoV-2 and SARS-CoV interfaces with ACE2

**Fig. 5:**
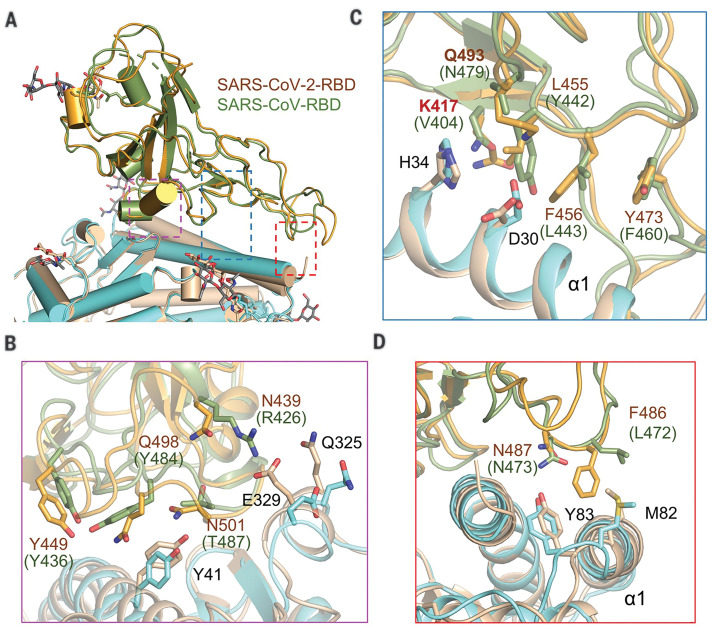
Interface comparison between SARS-CoV-2-RBD and SARS-CoV-RBD with ACE2. (**A**) Structural alignment for the SARS-CoV-2-RBD and SARS-CoV-RBD. The structure of the ACE2-PD and the SARS-CoV-RBD complex (PDB 2AJF) is superimposed on our cryo-EM structure of the ternary complex relative to the RBDs. The regions enclosed by the purple, blue, and red dashed lines are illustrated in detail in (B) to (D), respectively. SARS-CoV-2-RBD and the PD in our cryo-EM structure are colored orange and cyan, respectively; SARS-CoV-RBD and its complexed PD are colored green and gold, respectively. (**B** to **D**) Variation of the interface residues between SARS-CoV-2-RBD (labeled in brown) and SARS-CoV-RBD (labeled in green).

Superimposition of the RBD in the complex of SARS-CoV (SARS-CoV-RBD) and ACE2-PD [Protein Data Bank (PDB) 2AJF] with the RBD in our ternary complex shows that the SARS-CoV-2 RBD (SARS-CoV-2-RBD) is similar to SARS-CoV-RBD with a root mean square deviation (RMSD) of 0.68 Å over 139 pairs of Cα atoms (Fig. 5A) (*8*). Despite the overall similarity, a number of sequence variations and conformational deviations are found in their respective interfaces with ACE2 (Fig. 5 and fig. S8). At the N terminus of α1, the variations Arg^426^→Asn^439^, Tyr^484^→Gln^498^, and Thr^487^→Asn^501^ at equivalent positions are observed between SARS-CoV-RBD and SARS-CoV-2-RBD (Fig. 5B). More variations are observed in the middle of the bridge. The most prominent alteration is the substitution of Val^404^ in the SARS-CoV-RBD with Lys^417^ in the SARS-CoV-2-RBD. In addition, from SARS-CoV-RBD to SARS-CoV-2-RBD, the substitution of interface residues Tyr^442^→Leu^455^, Leu^443^→Phe^456^, Phe^460^→Tyr^473^, and Asn^479^→Gln^493^ may also change the affinity for ACE2 (Fig. 5C). At the C terminus of α1, Leu^472^ in the SARS-CoV-RBD is replaced by Phe^486^ in the SARS-CoV-2-RBD (Fig. 5D).

## Discussion

Although ACE2 is a chaperone for B^0^AT1, our focus is on ACE2 in this study. With the stabilization by B^0^AT1, we elucidated the structure of full-length ACE2. B^0^AT1 is not involved in dimerization, suggesting that ACE2 may be a homodimer even in the absence of B^0^AT1. Further examination suggests that a dimeric ACE2 can accommodate two S protein trimers, each through a monomer of ACE2 (fig. S9). The trimeric structure of the S protein of SARS-CoV-2 was recently reported, with one RBD in an up conformation and two in down conformations (PDB 6VSB) (*14*). The PD clashes with the rest of the S protein when the ternary complex is aligned to the RBD of the down conformation. There is no clash when the complex is superimposed on RDB in the up conformation, with a RMSD of 0.98 Å over 126 pairs of C_α_ atoms, confirming that an up conformation of RDB is required to bind to the receptor (fig. S9) (*14*).

Cleavage of the S protein of SARS-CoV is facilitated by cathepsin L in endosomes, indicating a mechanism of receptor-mediated endocytosis (*10*). Further characterization is required to examine the interactions between ACE2 and the viral particle as well as the effect of cofactors on this process (*25*, *33*). It remains to be investigated whether there is clustering between the dimeric ACE2 and trimeric S proteins, which may be important for invagination of the membrane and endocytosis of the viral particle, a process similar to other types of receptor-mediated endocytosis.

Cleavage of the C-terminal segment, especially residues 697 to 716 (fig. S4), of ACE2 by proteases, such as transmembrane protease serine 2 (TMPRSS2), enhances the S protein–driven viral entry (*34*, *35*). Residues 697 to 716 form the third and fourth helices in the neck domain and map to the dimeric interface of ACE2. The presence of B^0^AT1 may block the access of TMPRSS2 to the cutting site on ACE2. The expression distribution of ACE2 is broader than that of B^0^AT1. In addition to kidneys and intestine, where B^0^AT1 is mainly expressed, ACE2 is also expressed in lungs and heart (*27*). It remains to be tested whether B^0^AT1 can suppress SARS-CoV-2 infection by blocking ACE2 cleavage. Enteric infections have been reported for SARS-CoV, and possibly also for SARS-CoV-2 (*36*, *37*). B^0^AT1 has also been shown to interact with another coronavirus receptor, aminopeptidase N (APN or CD13) (*38*). These findings suggest that B^0^AT1 may play a regulatory role for the enteric infections of some coronaviruses.

Comparing the interaction interfaces of SARS-CoV-2-RBD and SARS-CoV-RBD with ACE2 reveals some variations that may strengthen the interactions between SARS-CoV-2-RBD and ACE2 and other variations that are likely to reduce the affinity compared with SARS-CoV-RBD and ACE2. For instance, the change from Val^404^ to Lys^317^ may result in a tighter association because of the salt bridge formation between Lys^317^ and Asp^30^ of ACE2 (Figs. 4C and 5C). The change from Leu^472^ to Phe^486^ may also result in a stronger van der Waals contact with Met^82^ (Fig. 5D). However, replacement of Arg^426^ with Asn^439^ appears to weaken the interaction by eliminating one important salt bridge with Asp^329^ on ACE2 (Fig. 5B).

Our structural work reveals the high-resolution structure of full-length ACE2 in a dimeric assembly. Docking the S protein trimer onto the structure of the ACE2 dimer with the RBD of the S protein bound suggests simultaneous binding of two S protein trimers to an ACE2 dimer. Structure-based rational design of binders with enhanced affinities to either ACE2 or the S protein of the coronaviruses may facilitate development of decoy ligands or neutralizing antibodies for suppression of viral infection.

## Supplementary Materials

science.sciencemag.org/content/367/6485/1444/suppl/DC1 Materials and Methods Figs. S1 to S9 Table S1 References (*39*–*52*) MDAR Reproducibility Checklist Movie S1

## Appendix

View/request a protocol for this paper from *Bio-protocol*.
